# Electronegative low-density lipoprotein increases the risk of ischemic lower-extremity peripheral artery disease in uremia patients on maintenance hemodialysis

**DOI:** 10.1038/s41598-017-04063-3

**Published:** 2017-07-05

**Authors:** Chiz-Tzung Chang, Ming-Yi Shen, An-Sean Lee, Chun-Cheng Wang, Wei-Yu Chen, Chia-Ming Chang, Kuan-Cheng Chang, Nicole Stancel, Chu-Huang Chen

**Affiliations:** 1 Division of Nephrology, China Medical University Hospital (CMUH), Taichung, Taiwan; 2 Cardiovascular Research Laboratory, CMUH, Taichung, Taiwan; 3 College of Medicine, China Medical University (CMU), Taichung, Taiwan; 4 Graduate Institute of Clinical Medical Science, CMU, Taichung, Taiwan; 5 Department of Medical Research, CMUH, Taichung, Taiwan; 6 Department of Medicine, Mackay Medical College, New Taipei, Taiwan; 7 Vascular and Medicinal Research, Texas Heart Institute, Houston, TX United States; 8 Lipid Science and Aging Research Center, Kaohsiung Medical University (KMU), Kaohsiung, Taiwan; 9 Center for Lipid Biosciences, KMU Hospital, Kaohsiung, Taiwan

## Abstract

Electronegative low-density lipoprotein (LDL) has been shown to increase coronary artery disease risk in hemodialysis patients, but its effect on the risk of peripheral artery disease (PAD) remains unclear. We separated plasma LDL from 90 uremia patients undergoing hemodialysis into 5 subfractions (L1–L5) according to charge by using fast-protein liquid chromatography with an anion-exchange column and examined the distribution of L5—the most electronegative LDL subfraction—in total LDL (i.e. L5%). During a 5-year period, we followed up with these patients until the occurrence of ischemic lower-extremity PAD. During the follow-up period, ischemic lower-extremity PAD developed in 24.4% of hemodialysis patients. L5% was higher in hemodialysis patients in whom ischemic lower-extremity PAD occurred (3.03% [IQR, 2.36–4.54], n = 22) than in hemodialysis patients in whom PAD did not occur (1.13% [IQR, 0.90–1.83], n = 68) (p < 0.001). Furthermore, L5% significantly increased the adjusted hazard ratio of ischemic lower-extremity PAD (1.54 [95% CI, 1.14–2.10]) (p = 0.005). Flow-mediated dilation was negatively associated with L5% (p < 0.001). Additionally, *in vivo* experiments from mice showed that L5 compromised endothelium-dependent vascular relaxation through a nitric oxide–related mechanism. Our findings indicate that increased L5% may be associated with the occurrence of ischemic lower-extremity PAD in hemodialysis patients.

## Introduction

Non-traumatic peripheral artery disease (PAD) is common among uremia patients, with an incidence up to 10-fold higher than that in non-uremia patients^1^. Lower-extremity PAD is a primary cause of mortality in uremia patients, as well the leading cause of amputation^2^. Results from the Dialysis Outcomes and Practice Pattern Study showed that the prevalence of PAD was 12% to 38% among different countries^3^, and the world-wide prevalence of PAD continues to increase^4^. The traditional risk factors associated with the pathogenesis of PAD are similar to those associated with systemic atherosclerosis, including age, smoking, diabetes, hypertension, and high body mass index (BMI)^5–8^. Serum hypercalcemia, hyperphosphatemia, and hyperparathyroidism are also important risk factors of PAD, especially in patients with chronic renal failure or uremia^9, 10^. Recently, genetic factors have also been found to be associated with the occurrence of PAD^11^.

Dyslipidemia, especially high levels of plasma cholesterol or low-density lipoprotein-cholesterol (LDL-C), is another traditional factor of atherosclerosis, but its role in the development of coronary artery disease in uremia patients remains controversial^12–14^. Similarly, the role of dyslipidemia in the development of PAD in uremia patients also remains unclear^15, 16^.

LDL is a heterogeneous substance composed of molecules differing in size, density, chemical composition, and electric charge. Importantly, LDL with increased electronegativity has been shown to be more atherogenic than its electropositive LDL counterpart^17^. We have previously shown that LDL can be separated into 5 subfractions (designated L1-L5) with increasing electronegativity by using fast-protein liquid chromatography with an anion-exchange column. L5, the most electronegative LDL subfraction^18^, has been studied extensively for its role in the pathogenesis of atherosclerosis and has been shown to be toxic to both cardiomyocytes and endothelial cells^19^. Furthermore, a high percentage of L5 in total LDL (ie, L5%) has been associated with acute myocardial infarction and coronary artery disease^20^. L5 from patients with increased cardiovascular risks such as diabetes and hypercholesterolemia decreases nitric oxide formation in endothelial cells, which impairs vascular relaxation and leads to endothelial dysfunction^21, 22^.

Recently, we showed that L5% is higher in patients with early chronic kidney disease or end-stage renal disease than in individuals with normal kidney function^23, 24^. Moreover, L5 stimulates platelet activation and enhances vascular thrombosis^25^, which promote ischemic atherosclerotic vascular disease formation. However,  the association between L5 and PAD in hemodialysis patients has not been explored. We conducted a cohort study to examine the occurrence of ischemic lower-extremity PAD among hemodialysis patients and estimated the hazard ratio of L5% on PAD.

## Results

### Increased L5% in hemodialysis patients with ischemic lower-extremity PAD

L5 distribution data were collected for 91 patients, but one patient was lost to follow-up 3 months after blood collection because of transferring to another dialysis center for maintenance hemodialysis therapy. Of the remaining 90 hemodialysis patients included in the study, ischemic lower-extremity PAD developed in 22 (24.4%) patients [PAD (+)] during the 5-year follow-up period but not in the other 68 patients [PAD (−)]. As shown in Table 1, the average follow-up time was shorter in the PAD (+) group than in the PAD (−) group. Patient age was higher in the PAD (+) group than in the PAD (−) group. Furthermore, PAD (+) patients had a higher percentage of diabetes mellitus (DM) and ischemic heart disease (IHD). Blood plasma levels of triglyceride, cholesterol, and LDL-C were also significantly higher in PAD (+) patients. Importantly, L5% and Hs-CRP levels were higher in the PAD (+) group than in the PAD (−) group. Other variables analyzed were not statistically different between groups (Table 1).

**Table 1:** Characteristics of patients with or without new onset ischemic lower-extremity peripheral artery disease during follow-up.

Variable	PAD (−) (n = 68)	PAD (+) (n = 22)	*P* value
Age (years)	52.5 (44.2–61.0)	59.5 (56.2–63.0)	0.015
Sex (male) (%)	84.3%	70.7%	0.15
HD Vintage (months)	51.0 (37.3–67.8)	42.0 (31.3–67.3)	0.17
BMI (kg/m^2^)	22.2 (21.2–24.0)	22.7 (21.3–26.0)	0.29
IHD (%)	29.4%	68.1%	<0.001
Hypertension (%)	76.4%	95.4%	0.06
DM (%)	10.3%	50.0%	0.001
Smoking (%)	13.2%	4.5%	0.44
Triglyceride (mg/dl)	134 (98–199)	256 (199–345)	<0.001
Cholesterol (mg/dl)	176 (150–192)	212 (176–224)	<0.001
HDL-C (mg/dl)	39 (34–44)	39 (31–48)	0.88
LDL-C (mg/dl)	98 (78–115)	111 (90–149)	0.021
L5%	1.13 (0.90–1.83)	3.03 (2.36–4.54)	<0.001
Hs-CRP (mg/dl)	0.34 (0.16–0.75)	1.18 (0.79–2.07)	<0.001
Calcium (mg/dl)	9.5 (9.1–10.1)	9.6 (9.2–10.2)	0.58
Phosphate (mg/dl)	5.2 (4.4–5.9)	5.6 (4.6–6.1)	0.22
Ca × P	49.2 (43.2–56.7)	52.5 (43.1–60.7)	0.33
iPTH (pg/mL)	198 (153–300)	247 (177–297)	0.35
BUN (mg/dl)	64.5 (58.2–72.5)	67.0 (56.2–75.2)	0.84
Creatinine (mg/dl)	10.2 (9.7–11.4)	10.8 (10.1–11.4)	0.46

Data are presented as the median value (interquartile range) or as the percentage. BMI = body mass index; BUN = blood urea nitrogen; Ca × P, calcium﻿-phosph﻿orous product; DM = diabetes mellitus; HD = hemodialysis; HDL-C = high-density lipoprotein-cholesterol; Hs-CRP = high sensitivity-C reactive protein; IHD = ischemic heart disease; iPTH = intact parathyroid hormone; LDL-C = low-density lipoprotein-cholesterol.

### The effect of L5% on the hazard ratio for the occurrence of ischemic lower-extremity PAD

To identify variables that enhance the risk of ischemic lower-extremity PAD, we used the Cox-proportional hazard method. Similar to other studies^4, 5, 7, 26^, we found that history of IHD, male sex, older age, DM, LDL-C levels, and BMI significantly increased the crude hazard ratio for the occurrence of ischemic lower-extremity PAD. However, when we adjusted for all of these factors, we found that only IHD and L5% significantly increased the hazard ratio for the occurrence of ischemic lower-extremity PAD (Table 2).

**Table 2:** Hazard ratios of different variables on ischemic lower-extremity PAD in hemodialysis patients.

Variable	Model 1		Model 2	
	HR (95% CI)	*P* value	HR (95% CI)	*P* value
L5%	1.75 (1.43–2.13)	<0.001	1.54 (1.14–2.10)	0.005
IHD	0.18 (0.07–0.48)	0.001	4.85 (1.59–14.76)	0.005
Cholesterol	1.02 (1.01–1.03)	<0.001	1.01 (0.99–1.03)	0.28
LDL-C	1.02 (1.01–1.04)	0.002	1.01 (0.99–1.04)	0.19
Hs-CRP	2.16 (1.34–3.47)	<0.001	0.90 (0.46–1.78)	0.51
DM	0.17 (0.07–0.44)	<0.001	1.36 (0.38–4.85)	0.63
Age	1.06 (1.01–1.11)	0.008	1.03 (0.98–1.10)	0.19
HD Vintage	0.98 (0.96–1.01)	0.079	—	—
Sex	0.40 (0.14–1.10)	0.076	—	—
Hypertension	0.19 (0.02–1.47)	0.11	—	—
Smoking	2.93 (0.39–21.85)	0.29	—	—
Calcium	1.11 (0.57–2.13)	0.76	—	—
Phosphate	1.20 (0.73–1.96)	0.46	—	—
BMI	1.15 (0.98–1.34)	0.075	—	—

Model 1 = crude hazard ratio; Model 2 = adjusted for L5%, IHD, LDL-C, Hs-CRP, DM, and age. BMI = body mass index; DM = diabetes mellitus; HD = hemodialysis; HR = hazard ratio; Hs-CRP = high-sensitivity C-reactive protein; IHD = ischemic heart disease; LDL-C = low-density lipoprotein-cholesterol; PAD = peripheral artery disease.

### Plasma L5% in patients with IHD history

We grouped patients according to IHD history or the presence of ischemic lower-extremity PAD. The median L5% (interquartile range) values of these subgroups are shown in Table 3. Patients with both IHD history and lower-extremity PAD had the highest median value of L5%, whereas patients without either IHD history or lower-extremity PAD had the lowest median value of L5%. The L5% of all patients with IHD history was 2.13% (1.21–2.80) (n = 35), whereas the L5% of all patients without IHD history was 1.18% (0.88–2.30) (n = 55). Thus, patients with IHD history had a significantly higher L5% than did patients without IHD history (p = 0.007).

**Table 3:** Plasma L5% of hemodialysis patients with or without IHD history and ischemic lower-extremity PAD.

	IHD history(−)	IHD history(+)
Ischemic lowerextremity PAD(−)	1.12% (0.86–1.63)(n = 48)	2.10% (1.12–2.61)(n = 20)
Ischemic lowerextremity PAD(+)	2.28% (2.16–3.78)(n = 7)	3.49% (2.43–5.14)(n = 15)

Data are shown as the median (interquartile range) L5%. IHD, ischemic heart disease; PAD, peripheral artery disease. (+) and (−) indicate the presence or absence, respectively.

### The effect of L5% on the hazard ratio for the occurrence of ischemic lower-extremity PAD in patients with IHD

We further used the Cox-proportional hazard method to identify variables that increased the risk of ischemic lower-extremity PAD in patients with IHD history. In patients with IHD history, the L5% was higher in patients who developed ischemic lower-extremity PAD during the follow-up period [2.49% (95% CI, 2.28–4.63) (n = 15)] than in patients who did not [1.25% (95% CI, 1.08–2.06) (n = 20)] (p < 0.001). Furthermore, L5%, cholesterol, and LDL-C levels significantly increased the crude hazard ratio for the occurrence of ischemic lower-extremity PAD in patients with IHD. When we adjusted for all of these factors, L5% was the only variable that significantly increased the hazard ratio for the occurrence of ischemic lower-extremity PAD in patients with IHD history (Table 4).

**Table 4:** Hazard ratios of different variables on ischemic lower-extremity PAD in hemodialysis patients with a history of IHD.

Variable	Model 1		Model 2	
	HR (95% CI)	*P* value	HR (95% CI)	*P* value
L5%	1.34 (1.03–1.75)	0.028	1.55 (1.01–1.86)	0.043
Cholesterol	1.02 (1.01–1.04)	0.003	1.00 (0.99–1.04)	0.09
LDL-C	1.03 (1.02–1.05)	0.032	1.01 (0.98–1.04)	0.45
DM	0.41 (0.13–1.30)	0.13	—	—
Hs-CRP	1.48 (0.76–2.89)	0.24	—	—
Age	0.98 (0.93–1.04)	0.61	—	—
HD vintage	0.98 (0.96–1.01)	0.13	—	—
Sex	0.52 (0.18–1.47)	0.93	—	—
Hypertension	0.39 (0.04–3.21)	0.09	—	—
Smoking	3.47 (0.46–25.98)	0.34	—	—
Calcium	1.60 (0.74–2.57)	0.58	—	—
Phosphate	1.20 (0.65–2.19)	0.55	—	—
BMI	0.98 (0.83–1.17)	0.89	—	—

Model 1 = crude hazard ratio; Model 2 = adjusted for L5%, cholesterol, and LDL-C. BMI = body mass index; DM = diabetes mellitus; HD = hemodialysis, HR = hazard ratio; Hs-CRP = high-sensitivity C-reactive protein; LDL-C = low-density lipoprotein-cholesterol; PAD = peripheral artery disease.

### Kaplan-Meier analysis using an L5% cutoff value for ischemic lower-extremity PAD

**Figure 1:**
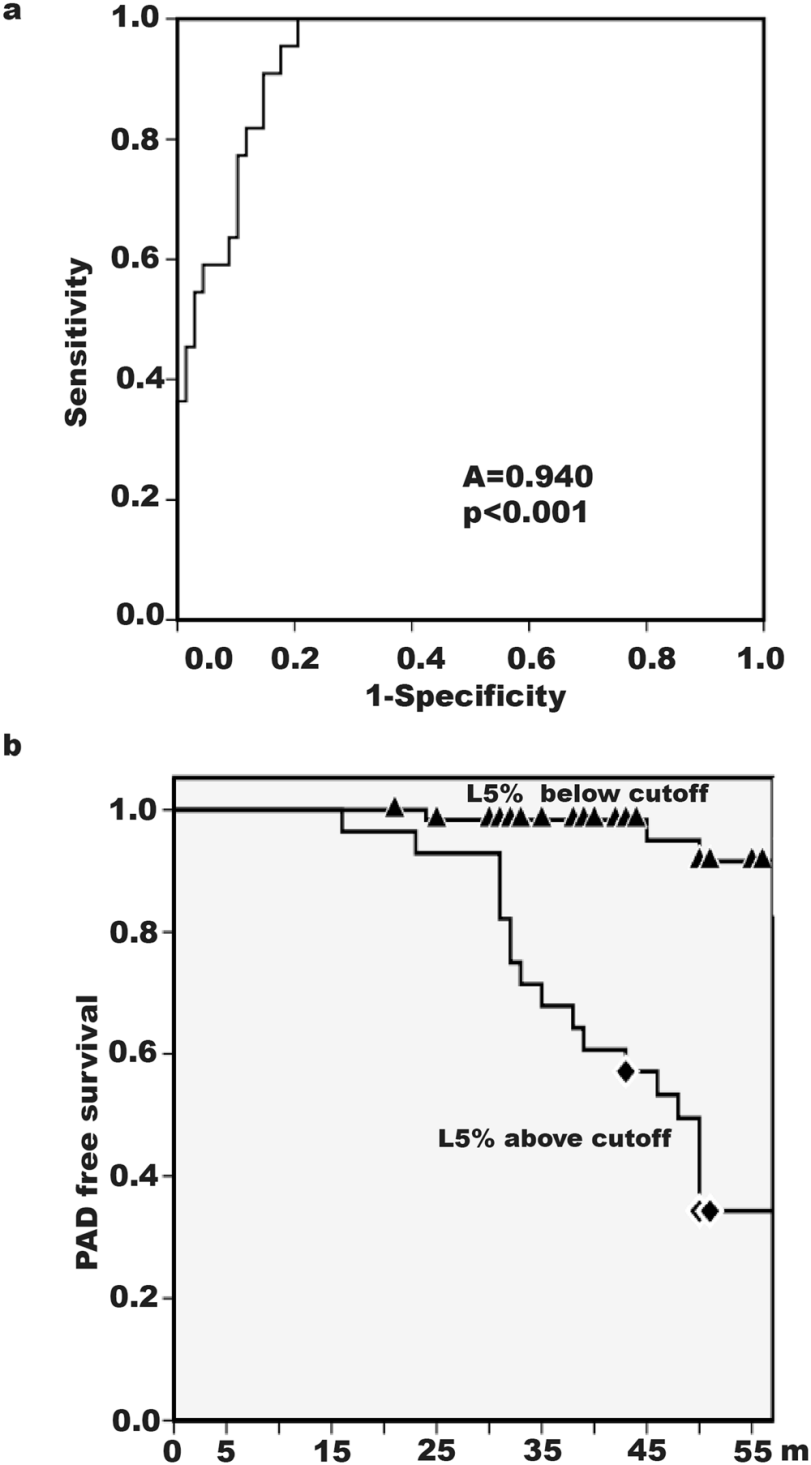
Receiver operating characteristic (ROC) analysis and ischemic lower-extremity peripheral arterial disease (PAD)-free survival in hemodialysis patients. (**a**) ROC analysis showed an area under the curve of 0.940 (p < 0.001). An L5% cutoff value of 2.239 was associated with ischemic lower-extremity PAD with a sensitivity of 0.909 and a specificity of 0.853. A = area under curve. (**b**) Patients with an L5% below 2.239 had a higher ischemic lower-extremity PAD-free survival rate than did patients with an L5% above 2.239 (log-rank test, p < 0.001).

To find the best cutoff value for ischemic lower-extremity PAD, we used receiver operating characteristic analysis. The area under the curve was 0.940 (95% CI, 0.895–0.985) (p < 0.001). When we set the L5% cutoff value at 2.239, we observed a sensitivity of 0.909 and a specificity of 0.853 for ischemic lower-extremity PAD (Fig. 1a).

When we grouped all 90 patients according to whether their L5% was above or below the cutoff value, Kaplan-Meier analysis showed that the ischemic lower-extremity PAD–free survival rate in patients with an L5% below 2.239 was significantly higher than that in patients with an L5% above 2.239 (p < 0.001, log-rank test) (Fig. 1b).

### Positive association between L5% and Hs-CRP levels in hemodialysis patients

Because we found that age, IHD, DM, and levels of triglyceride, cholesterol, LDL-C, and Hs-CRP were significantly higher in the PAD (+) group than in the PAD (−) group, we performed linear regression analysis to characterize the association of L5% with these variables in hemodialysis patients. In univariate regression analysis, IHD, DM, and levels of triglyceride, cholesterol, LDL-C, and Hs-CRP were significantly associated with L5%. In multivariate regression analysis in which we adjusted for all of these factors, only Hs-CRP level was positively associated with L5% (p = 0.004) (Table 5).

**Table 5:** Univariate and multivariate linear regression analysis for identifying independent factors associated with L5% in hemodialysis patients.

Variable	Univariate		Multivariate	
	B (SE)	*P* value	B (SE)	*P* value
Age	0.013 (0.013)	0.33	−0.002 (0.012)	0.85
IHD	0.624 (0.298)	0.039	0.166 (0.284)	0.56
DM	1.257 (0.280)	<0.001	0.479 (0.304)	0.12
Triglyceride	0.007 (0.001)	<0.001	0.002 (0.002)	0.26
Cholesterol	0.015 (0.004)	<0.001	0.010 (0.006)	0.10
LDL-C	0.013 (0.005)	0.018	−0.007 (0.008)	0.37
Hs-CRP	0.989 (0.187)	0.001	0.611 (0.204)	0.004
IHD	0.624 (0.298)	0.039	0.166 (0.284)	0.56

DM = diabetes mellitus; Hs-CRP = high-sensitivity C-reactive protein; IHD = ischemic heart disease; LDL-C = low-density lipoprotein-cholesterol; B = regression coefficient; SE = standard error.

### Negative correlation of flow-mediated dilation (FMD) with L5% and decreased FMD in patients with ischemic lower-extremity PAD

Age, IHD, DM, smoking, hypertension, dyslipidemia or hypercholesterolemia, hyperphosphotemia, and high Hs-CRP levels are factors associated with FMD^27–33^. We performed univariate regression analysis and found that L5%, IHD, DM, and cholesterol levels were significantly associated with FMD. However, in multivariate regression analysis, only L5% was significantly associated with FMD (p < 0.001) (Table 6). The median value of FMD in the PAD (+) group was 4.44% (IQR, 3.10–5.22), whereas the median FMD value in the PAD (−) group was 6.10% (IQR, 5.22–8.30). Thus, the median value of FMD was significantly lower in PAD (+) hemodialysis patients than in PAD (−) hemodialysis patients (p < 0.001).

**Table 6:** Univariate and multivariate linear regression analysis for identifying independent factors associated with FMD in hemodialysis patients.

Variable	Univariate		Multivariate	
	B (SE)	*P* value	B (SE)	*P* value
L5%	−1.016 (0.155)	<0.001	−0.992 (0.191)	<0.001
Age	−0.015 (0.024)	0.52	−0.004 (0.023)	0.85
IHD	−1.535 (0.512)	0.004	−0.918 (0.498)	0.07
DM	−1.321 (0.529)	0.014	−0.073 (0.533)	0.90
Smoking	−0.957 (0.827)	0.25	−0.874 (0.002)	0.24
Hypertension	−0.248 (0.669)	0.71	0.289 (0.598)	0.63
Cholesterol	−0.021 (0.006)	0.001	−0.006 (0.006)	0.37
Phosphate	−0.541 (0.283)	0.06	−0.237 (0.255)	0.36
Hs-CRP	−0.658 (0.370)	0.08	0.639 (0.379)	0.56

DM = diabetes mellitus; FMD = flow-mediated dilation; Hs-CRP = high-sensitivity C-reactive protein; IHD = ischemic heart disease; B = regression coefficient; SE = standard error.

### L5-induced decrease in vascular relaxation through a phospho-eNOS–related mechanism

**Figure 2:**
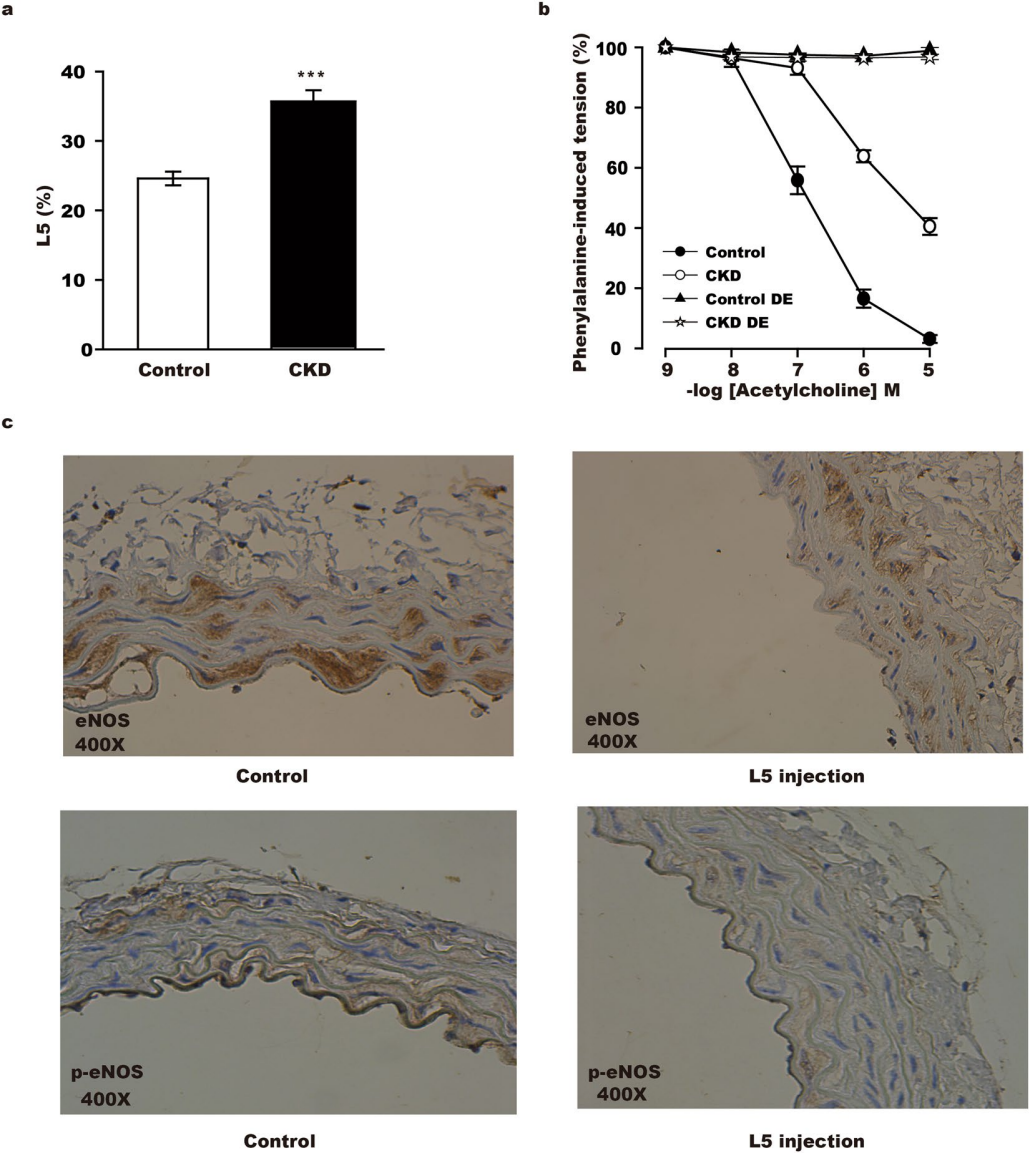
L5-induced decrease in vascular relaxation through a phospho-eNOS–related mechanism (**a**) The plasma L5 level (L5%) was higher in CKD rats than in control rats (35.7 ± 1.6% vs. 24.6. ± 1.0%, n = 6 per group) (***p < 0.001). (**b**) Phenylalanine-induced aortic tension, which is reversed by the addition of acetylcholine, was lower in control rats (open circle, n = 6) than in CKD rats (black circle, n = 6). Aortic tension is presented as the difference in area under the curve (AUC) (AUC open circle vs. AUC black circle, p < 0.001). The ability of acetylcholine to reverse phenylalanine-induced aortic tension was abolished in both control rats (black triangle, n = 6) and CKD rats (empty star, n = 6) when the aortic endothelium was denuded (DE) (AUC black triangle vs. AUC empty star, p = 0.10). The non-parametric method of integrated AUC measure was used to compare groups. (**c**) Representative immunohistochemistry results show that the expression of eNOS and phospho-eNOS (p-eNOS) in the aortic endothelium was lower in mice injected with L5 (n = 4, right panel) than in mice injected with normal saline (n = 4, left panel).

L5% was higher in rats with chronic kidney disease (CKD) induced by 5/6 nephrectomy than in control rats (Fig. 2a). When aortas with intact endothelium from CKD rats and control rats were subjected to an *ex vivo* tension study, aortas from CKD rats showed an impaired acetylcholine-induced relaxation response (Fig. 2b). However, the acetylcholine-induced relaxation response was abolished in both control and CKD rats when the vascular endothelium was denuded (Fig. 2b). When we treated C57BL/6 mice with chronic venous injections of L5 or normal saline, the expression of both eNOS and phospho-eNOS protein was lower in the aortic endothelium of L5-treated mice than in that of control mice (Fig. 2c).

## Discussion

In our study, we showed that L5% was significantly higher in hemodialysis patients in whom ischemic lower-extremity PAD developed than in hemodialysis patients in whom ischemic lower-extremity PAD did not develop and that L5% increased the hazard ratio for the occurrence of ischemic lower-extremity PAD in this patient population. The positive association of L5% with ischemic lower-extremity PAD was observed in hemodialysis patients, regardless of whether they had a history of IHD, although our findings indicated that hemodialysis patients with IHD history had a higher risk of ischemic lower-extremity PAD than did hemodialysis patients without IHD. Furthermore, we identified a negative correlation of L5% with FMD. Our findings suggest that L5% may be associated with the occurrence of PAD in uremia patients on hemodialysis.

We found that the percentage of ischemic lower-extremity PAD was higher in hemodialysis patients with older age, DM, IHD history, or hyperlipidemia—all traditional risk factors for PAD in non-uremia patients. However, no difference was seen between hemodialysis patients who were smokers or non-smokers or between patients with or without hypertension. Compared to the general adult population, the prevalence of smokers in our cohort of hemodialysis patients was low, whereas the prevalence of hypertension was high, which may explain these findings.

History of IHD is an important risk factor of PAD^34^. The results of our Cox-regression analysis showed that L5% and IHD history had a significantly higher hazard ratio for the occurrence of ischemic lower-extremity PAD than did all other traditional risk factors for PAD. Furthermore, L5% was higher in patients with a history of IHD than in patients without a history of IHD. In our subgroup analysis among patients with IHD history, L5% was higher in the PAD (+) group than in the PAD (−) group. These results suggest that L5% has a high hazard ratio for ischemic lower-extremity PAD, even in IHD patients.

There are several possible mechanisms that may underlie L5-mediated endothelial dysfunction and PAD occurrence in hemodialysis patients. L5 LDL contains apolipoprotein B and is rich in apolipoprotein CIII and triglycerides^18, 24^. Similar to other LDL that is rich in apolipoprotein CIII or triglycerides, L5 is highly atherogenic^35, 36^. Our previous studies have shown that plasma L5 levels are increased in patients with high cardiovascular disease risks, such as patients with familial hypercholesterolemia or diabetes and in smokers and hemodialysis patients^18, 21, 24, 37^. Furthermore, L5 from hypercholesterolemic patients was shown to enhance the production of CRP by endothelial cells via the activation of lectin-like oxidized LDL receptor-1 (LOX-1)^38^. CRP is a marker of systemic inflammation and also of PAD^39^. CRP has been shown to impair endothelial-dependent vasodilatation^40^ and to enhance LOX-1 expression through FcγRs (Fc-gamma receptors), which can further compromise endothelial function^41^. Consistent with the results of the study with L5 from hypercholesterolemic patients^38^, we found that L5% was positively associated with Hs-CRP levels in hemodialysis patients with PAD, indicating that increased Hs-CRP levels may be a possible mechanism underlying L5-mediated endothelial dysfunction and PAD occurrence in hemodialysis patients.

In addition, L5 can induce endothelial cell apoptosis through LOX-1 and reduce the formation of nitric oxide in endothelial cells, which leads to endothelial dysfunction^21^. Similarly, through LOX-1, L5 can activate platelet activation and promote thrombogenesis^25^. Both endothelial dysfunction and enhanced thrombogenesis predispose patients to atherosclerotic vascular disease including PAD^42^. Endothelial cell dysfunction can be manifested by poor FMD^24^. Poor FMD is a marker of atherosclerotic vascular disease^43, 44^ and can also predict an atherosclerotic vascular event^28^. In our multivariate regression analysis, L5% was significantly associated with poor FMD and a high cumulative rate of ischemic lower-extremity PAD, whereas traditional risk factors were not (Table 6). Previously, we showed that L5 can inhibit eNOS phosphorylation in human aortic endothelial cells and impair vascular relaxation via a nitric oxide–mediated mechanism. Therefore, through the suppression of phospho-eNOS in endothelial cells, L5 impaired vascular relaxation and resulted in a decrease in FMD^24^. In the present study, we showed that the expression of both eNOS and phospho-eNOS protein was suppressed in endothelial cells of the terminal aorta in mice that received chronic venous injections of L5 (Fig. 2c). We used the terminal portion of the aorta in place of arteries in the extremities because its location and size made it easier to retrieve than vessels in the limbs of mice.

Compatible with the negative correlation that we observed between L5 and FMD, L5 has been associated with oxidative stress^45^. Previous studies have shown that oxidative stress is associated with PAD in hemodialysis patients. Reducing oxidative stress in these patients with propionyl L-carnitine can significantly increase nitric oxide formation, decrease vasoconstrictive endothelin-1 production, and improve vascular hemodynamic flow^46, 47^. Together, these findings suggest that the association between L5% and lower-extremity PAD in uremia patients may in part be attributed to the promotion of oxidative stress by L5 LDL.

Lipid-lowering agents have been shown to have anti-inflammatory effects and protective effects on endothelial function^48, 49^. Previously, studies in which lipid-lowering agents were examined for their ability to lower cardiovascular mortality in hemodialysis patients showed no efficacy, even though serum cholesterol and LDL-C levels were successfully decreased by these agents^12, 13^. Therefore, lipid-lowering agents that specifically target the lowering of L5% may be more important than cholesterol-lowering in the prevention of atherosclerotic vascular disease.

A limitation of our study was the sample size. Because 20 ml of blood are required to measure L5%, in addition to another 20 ml of blood for other laboratory tests, recruiting patients to donate blood for the LDL distribution study can be challenging. However, because this was a cohort study with a 5-year duration of observation, the study design, in part, compensated for the small sample size. Furthermore, all but one of our patients for whom L5 distribution data were collected completed the study. We included only patients who had been receiving hemodialysis at our center for more than 6 months, and only the one patient who was lost to follow up transferred to another dialysis center. Notably, at our hospital, the rate of kidney transplantation is lower than 2% per year.

We found no difference in the severity of vascular calcification between PAD (+) and PAD (−) groups. In addition, serum phosphate, calcium, or intact parathyroid hormone levels, which are known to contribute to vascular calcification when elevated^10, 50^, did not increase the hazard ratios for ischemic lower-extremity PAD in our hemodialysis patients. However, our study did not contain sufficient data to rule out the possibility that lower extremity artery calcification resulted in PAD. We also did not score coronary artery or abdominal aorta calcifications with computed tomography.

In conclusion, ischemic lower-extremity PAD is common in hemodialysis patients, and our study findings indicate that increased levels of L5% may be associated with its occurrence in this patient population. Furthermore, our findings suggest that hemodialysis patients with or without IHD are both at risk of ischemic lower-extremity PAD in the presence of high L5 levels in the blood.

## Methods

### Patients

Our study was an observational cohort study, which included 90 adult patients with uremia who underwent maintenance hemodialysis therapy twice a week for at least 6 months at China Medical University Hospital (CMUH). The study was performed according to the STROBE guidelines for observational studies. Hemodialysis therapy and blood collection were performed after written, informed patient consent was obtained. This study protocol was approved by the CMUH institutional review board (reference number: CMUH104-REC2-160) and adhered to the Declaration of Helsinki.

Ischemic lower-extremity PAD was defined as intermittent claudication, rest pain of the feet or sole, lower-leg tissue loss from an arterial insufficiency ulcer, or gangrene of the feet or sole. The diagnosis criteria for PAD followed the Fontaine Classification, and we accepted patients only with stage II, III, and IV PAD^51^. The follow-up of recruited patients started from the day of blood collection and continued until ischemic lower-extremity PAD or mortality occurred. Our follow-up period was from January 1, 2010 to December 31, 2015. We excluded patients with pre-existing ischemic PAD and patients who were lost to follow-up because of transferring to another dialysis center for dialysis therapy within 6 months of blood sampling for the L5 distribution study.

For all patients included in the study, we obtained a complete medical history and routine laboratory test results on the day of blood collection. Medical history included smoking, hypertension, DM, and previous IHD. The diagnosis of IHD was made by a cardiologist on the basis of clinical symptoms and imaging studies such as myocardial scintigraphy or coronary angiography. Routine laboratory tests were performed with mid-week predialysis fasting venous blood and included measurements of serum calcium, phosphate, blood urea nitrogen, creatinine, cholesterol, triglyceride, LDL-C, high-density lipoprotein-cholesterol, intact parathyroid hormone (iPTH), and high-sensitivity C-reactive protein (Hs-CRP).

### Isolation of L5

Blood was separated into red blood cells and plasma immediately after collection, and LDL was isolated from plasma with ultracentrifugation^24^. The isolated LDL was then loaded in a fast-protein liquid chromatography machine with an anion-exchange column. LDL subfractions L1 to L5 were eluted sequentially, as previously described^18^.

### Diagnosis of ischemic lower-extremity PAD

Ischemic lower-extremity PAD was diagnosed by using the patient’s ischemic pain history and the ankle-brachial index (ABI) test. ABI measurements were performed by using an ABI-form device (Colin VP1000; Omron Healthcare, Inc., Kyoto, Japan), with the patient fully resting in a supine position. PAD was defined by having an ABI value less than 0.9^52^. In all patients, the PAD diagnosis and the site of vascular occlusion were further confirmed by using duplex sonography.

### Flow-mediated dilation measurement

FMD was measured on the day of blood collection by inflating a sphygmomanometer cuff around the mid-forearm to 250 mmHg. The cuff was then deflated 5 min later. The diameter of the brachial artery was measured after cuff inflation and deflation from a B-mode ultrasound image (Logiq e, GE Healthcare, Wauwatosa, WI, USA). FMD was defined as the percent increase of brachial artery diameter after cuff deflation^53^.

### Aortic ring tension assay

All animal studies adhered to the National Institutes of Health *Guide for the Care and Use of Laboratory Animals Eighth Edition*. All animal study protocols were approved by Institutional Animal Use and Care Committee (protocol number: 2016393). Twelve 2-month-old Sprague–Dawley rats were used in this study; 6 underwent 5/6 nephrectomy to induce CKD as previously described^54^, whereas 6 control rats did not. Three months later, rats were euthanized, blood was collected, and the thoracic aorta was excised. L5 was isolated, and aortas were dissected into four 3-mm-long rings. We recorded the vascular tension by using a data acquisition system (PowerLab, ADInstruments Ltd., Denver, CO, USA). Increasing concentrations of acetylcholine (10 nM–10 μM) were applied to the rings during the sustained phase (considered as 100%) of phenylephrine (0.3 μM)-induced aortic ring contraction. In some aortic ring preparations, endothelium was denuded by using a cotton swab as previously described^24^.

### Immunohistochemistry

Two-month-old C57BL/6 male mice received daily venous injections of L5 (2 mg/kg)(n = 4) or normal saline (control)(n = 4) for one month. At the end of the experiment, mice were euthanized, and the abdominal aorta was excised and fixed in 10% formalin. The terminal part of the abdominal aorta was cut into pieces and embedded in paraffin. Immunohistochemistry was performed by incubating aortic sections with mouse anti-eNOS antibody (1:50) (Abcam, Cambridge, MA, USA) or mouse anti–phospho-eNOS antibody (1:50) (Santa Cruz Biotechnology, Santa Cruz, CA, USA) at 4 °C overnight and then with horseradish peroxidase-conjugated secondary antibody (1:500) (Santa Cruz Biotechnology) at room temperature. Images were captured by using a Nikon E600 light microscope (Nikon, Kokyo, Japan; magnification, 400X).

### Statistical analysis

Univariate analysis of quantitative variables was performed by using the Mann-Whitney U test, and data were expressed as the median value with the interquartile range (IQR, 25%–75%). For categorical variables, the chi-square test or Fisher’s exact test was used. The Cox-proportional hazard method was used to estimate the hazard ratio with a 95% CI for the development of ischemic low-extremity PAD after adjusting for covariate factors. Receiver operating characteristic analysis was performed to find the best cutoff value of L5% for ischemic lower-extremity PAD. Univariate and multivariate linear regression studies were performed to identify factors significantly associated with L5% and FMD. All calculations were performed by using IBM SPSS Statistics version 21.0 for Mac (SPSS Inc., Chicago, IL, USA), and all statistical analyses were reviewed by a biostatistician.
